# Supplementary material for: Residents need competence not confidence: A retrospective evaluation of the new competency education program for Korean neurology residents
Source: PLoS One. 2023 Oct 5;18(10):e0290503. doi: 10.1371/journal.pone.0290503 (PMC10553350; doi:10.1371/journal.pone.0290503)
Supplement: S1 Appendix — It is an assessment tool that measures residents’ competence in their ability to perform 13 critical clinical tasks (competencies-Communication, Collaboration, Clinical knowledge, independence, Community support and understanding care System). (DOCX) [file pone.0290503.s001.docx]

**Appendix 1. K-NEPA 13 stands for Korea Neurologist’s Entrustable Professional Activities 13. It is an assessment tool that measures residents' competence in their ability to perform 13 critical clinical tasks (competencies-Communication, Collaboration, Clinical knowledge, independence, Community support and understanding care *System*)**

| 1. Accurately communicate the patient's neurological condition to the patient and caregivers and discuss treatment options together (**Communication**) 2. Can educate and manage patients with rare neurological diseases and their families (**Communication**) 3. Effectively collaborate with other medical staffs (**Collaboration**) 4. Efficiently perform history taking and accurate neurological examination according to the level of consciousness (**Clinical Knowledge**) 5. Localize lesions by the results of neurological examination. (**Clinical Knowledge**) 6. Differentiate and treat common neurological abnormalities. (**Clinical Knowledge**) 7. Appropriately respond to neurological emergencies and manage critical-condition patients (**Clinical Knowledge**) 8. Accurately perform or interpret neurological tests (**Clinical Knowledge**) 9. Independently perform outpatient clinic (**Clinical Knowledge**) 10. Supervise and provide feedback to junior residents (**Independence**) 11. Improve the quality of care by self-learning and searching for new research outcomes (**Independence**) 12. Carry out education on the prevention and management of neurological diseases (**Community Support**) 13. Understand and use various care support systems for patients with dementia and patients with chronic neurological diseases (**System**) |
| --- |
