# Supplementary material for: Residents need competence not confidence: A retrospective evaluation of the new competency education program for Korean neurology residents
Source: PLoS One. 2023 Oct 5;18(10):e0290503. doi: 10.1371/journal.pone.0290503 (PMC10553350; doi:10.1371/journal.pone.0290503)
Supplement: S2 Appendix — (DOCX) [file pone.0290503.s002.docx]

**Appendix 2. The survey items (8) used in this study**

| 1. "Please rank the following competencies from the most important to the least information in your residency program:  - Clinical knowledge - Communication with patients/caregivers - Collaboration with peers - Professionalism and ethical concerns  1. Please select the options that best represent your primary sources of acquiring each competency. Choose from the following options  - Formal lectures and workshops - Supervisory guidance and feedback - Self-study and independent learning - Interactions with senior residents - Interactions with fellow residents - Simulation-based training - Other (please specify)"   1. Please indicate your perceived level of preparedness in dealing with challenging clinical scenarios that require the application of core competencies (clinical knowledge, communication with patients/caregivers, collaboration with peers, and ethical concerns) in your current level of training (note that each competency was separately asked)." - Not prepared at all - Slightly prepared - Moderately prepared - Very prepared - Extremely prepared  1. When making clinical decisions, to what extent do you actively seek out information that challenges your existing beliefs or thoughts?  - Not active at all - Slightly active - Moderately active - Very active - Extremely active  1. What makes you not actively seek out information that challenges your existing beliefs and thoughts (note that this question was administered only for those who answered “Not active” or “slightly active” at Problem 7)?  - That often never works - That is too busy - That is too complicated - That is what we have always done - Other (please specify) |
| --- |
